# Mitochondrial DNA Deficiency and Supplementation in *Sus scrofa* Oocytes Influence Transcriptome Profiles in Oocytes and Blastocysts

**DOI:** 10.3390/ijms24043783

**Published:** 2023-02-14

**Authors:** Takashi Okada, Stephen McIlfatrick, Justin C. St. John

**Affiliations:** Mitochondrial Genetics Group, Robinson Research Institute, School of Biomedicine, Faculty of Health and Medical Sciences, The University of Adelaide, Adelaide, SA 5000, Australia

**Keywords:** mitochondrial DNA, mitochondrial supplementation, blastocyst, oocyte, *Sus scrofa*, transcriptome analysis, Brilliant Cresyl Blue, RNA metabolism, meiosis, OXPHOS

## Abstract

Mitochondrial DNA (mtDNA) deficiency correlates with poor oocyte quality and fertilisation failure. However, the supplementation of mtDNA deficient oocytes with extra copies of mtDNA improves fertilisation rates and embryo development. The molecular mechanisms associated with oocyte developmental incompetence, and the effects of mtDNA supplementation on embryo development are largely unknown. We investigated the association between the developmental competence of *Sus scrofa* oocytes, assessed with Brilliant Cresyl Blue, and transcriptome profiles. We also analysed the effects of mtDNA supplementation on the developmental transition from the oocyte to the blastocyst by longitudinal transcriptome analysis. mtDNA deficient oocytes revealed downregulation of genes associated with RNA metabolism and oxidative phosphorylation, including 56 small nucleolar RNA genes and 13 mtDNA protein coding genes. We also identified the downregulation of a large subset of genes for meiotic and mitotic cell cycle process, suggesting that developmental competence affects the completion of meiosis II and first embryonic cell division. The supplementation of oocytes with mtDNA in combination with fertilisation improves the maintenance of the expression of several key developmental genes and the patterns of parental allele-specific imprinting gene expression in blastocysts. These results suggest associations between mtDNA deficiency and meiotic cell cycle and the developmental effects of mtDNA supplementation on *Sus scrofa* blastocysts.

## 1. Introduction

The mitochondrial genome (mtDNA) is a double stranded circular DNA that is approximately 16.6 kb in size and encodes 13 of the subunits of the electron transfer chain, which generates the vast majority of cellular ATP through oxidative phosphorylation (OXPHOS) [1]. It also encodes 2 rRNAs and 22 tRNAs and has one major non-coding region, the D-Loop, that is the site of interaction for the nuclear-encoded transcription and replication factors that translocate to the mitochondrion to drive first transcription and then replication [2]. Furthermore, mtDNA is inherited from the population present in the oocyte at the time of fertilisation and is, thus, a maternally-only inherited genome [3].

In human and other mammalian species, including the pig, there are associations between mtDNA copy number, low levels of ATP content, and the ability of the oocyte to fertilise and give rise to embryos [4,5,6,7,8]. For example, oocytes with low mtDNA are more likely to fail to fertilise or arrest during preimplantation development. Indeed, mitochondrial deficiency can lead to dysfunction in a number of cell types [9,10]. In the oocyte, mtDNA deficiency appears to be one of the causes of female factor infertility [4,5,8]. To overcome this problem, several approaches have been proposed and adopted. For example, oocytes can be supplemented with either ooplasm [11,12] or purified populations of mitochondria from autologous or heterologous sources. These sources can include sister (derived from the same ovary) or donor (third party) oocytes, cumulus cells and egg precursor cells [13,14,15,16].

The supplementation of oocytes at the time of fertilisation with mtDNA, packaged within mitochondria derived from sister oocytes, has shown to improve the rates of the fertilisation and development of mammalian embryos [13,14,17,18]. This process adds approximately an extra 800 copies of mtDNA [13], which represents 0.3% of the fertilisable oocyte’s total mtDNA content (~300,000). Consequently, this process is unlikely to increase mitochondrial bioenergetics. Instead, in mtDNA-deficient oocytes, it triggers an mtDNA replication event whereby mtDNA copy number is increased by 4.4-fold by the 2-cell stage of embryo development and increases the oocyte’s potential to develop to the blastocyst stage, the final stage of preimplantation development [13]. The induction of early mtDNA replication in mtDNA-deficient oocytes could improve embryo quality by stabilising the embryo prior to embryonic genome activation [13]. Indeed, we have shown that mtDNA supplementation alters the DNA methylation profile of over 2000 nuclear genomic regions in blastocysts and influenced the expression of a subset of nuclear-encoded genes [13,19,20]. However, it is still not known how mtDNA supplementation affects the developmental transition from the oocyte to blastocyst at the transcript level, such as the expression profiles of key developmental and imprinted genes.

**Figure 1:**
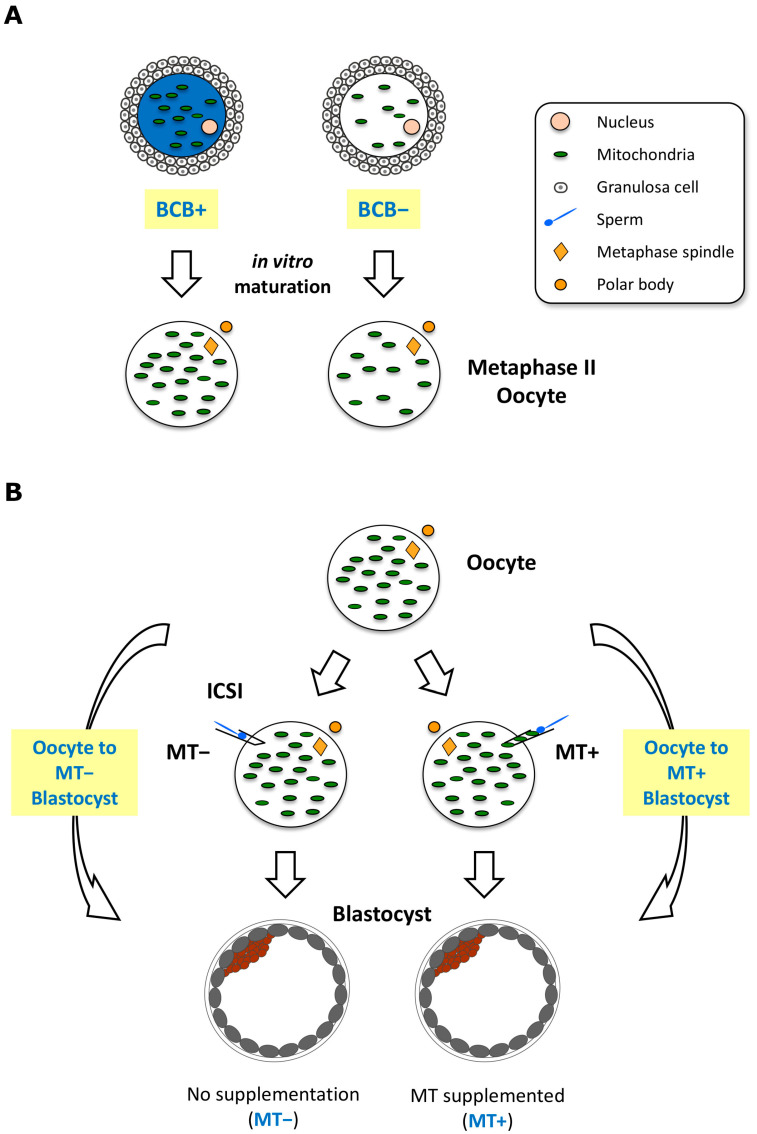
Schematic representation of the materials used for RNAseq and data analysis. (**A**) Brilliant Cresyl Blue (BCB) labelling of cumulus-oocyte-complexes to assess cytoplasmic maturity of oocytes. After labelling of oocytes, BCB+ and BCB− oocytes were washed and cultured separately. Denuded metaphase II oocytes were pooled and used for RNAseq. (**B**) Schematic representation of the mtDNA supplementation procedure, i.e., fertilisation by intracytoplasmic sperm injection (ICSI) with (MT+) or without (MT−) extra copies of mtDNA. RNAseq data from BCB+ oocytes and individual blastocysts generated with or without mtDNA supplementation were used to perform longitudinal differential gene expression (DEG) analysis by comparing the transition from oocyte to either MT− or MT+ blastocysts. Details of the procedure to undertake the analysis are found in the Materials and Methods.

One approach to distinguish between oocytes with low and high mtDNA copy number is to label oocytes once harvested from ovaries with Brilliant Cresyl Blue (BCB) and prior to in vitro culture to the metaphase II stage. This provides a non-invasive visual assessment of oocyte quality and developmental competence [14,21,22,23]. The use of BCB determines the intracellular levels of glucose-6-phosphate dehydrogenase (G6PDH), an indicator of developmental competence. G6PDH activity is high in the developing, immature oocyte and breaks down the label, resulting in a colourless cytoplasm (BCB negative, BCB−). On the other hand, oocytes that have completed their growth phase have low levels of G6PDH activity, are unable to break down the label, and exhibit a blue cytoplasm (BCB positive, BCB+) (Figure 1A). BCB labelling is associated with fertilization outcome and blastocyst formation rates [14,21,24]. Developmentally incompetent, BCB− oocytes usually fail to mature to the metaphase II stage, exhibit small cytoplasmic volumes, low levels of mtDNA copy number and mitochondrial activity, and changes in gene expression [13,14,21,22,23,24,25], indicative of insufficient cytoplasmic maturation [26,27]. BCB+ oocytes more readily reach the metaphase II stage, fertilise, have higher mtDNA copy number, larger cytoplasmic volumes, and express genes more indicative of a fertilisable oocyte. Nevertheless, some BCB− oocytes progress to the metaphase II stage after in vitro maturation [14,21,28] and appear to have cytoplasmic volumes similar to BCB+ oocytes [24]. However, they have significantly fewer copies of mtDNA and are, thus, indicative of mtDNA deficiency [13]. Consequently, these metaphase II oocytes derived from BCB− cumulus-oocyte complex exhibit discordant nuclear and cytoplasmic maturation [27,29]. Therefore, the molecular analysis of accumulated transcripts in BCB+ and BCB− oocytes at the metaphase II stage would help to understand the underlying mechanisms of developmental incompetence associated with mtDNA deficiency.

We have previously shown that genes associated with mtDNA replication are downregulated in BCB− porcine oocytes [30] and that there are global differences in gene expression between BCB+ and BCB− oocytes at the metaphase II stage, though this analysis was restricted by limited annotation of older pig genome assemblies [31,32]. Recently, single-cell RNAseq analysis of BCB+/− porcine germinal vesicle (GV) stage oocytes identified 155 differentially expressed genes and highlighted the downregulation of the *cell division cycle 5-like protein* (*CDC5L*) gene in BCB− oocytes [33]. Indeed, inhibition of CDC5L by siRNA and antibody microinjection impaired porcine oocyte maturation. More recently, BCB stained bovine oocytes at the mature metaphase II stage have been investigated by single-cell RNAseq, but there were no significant differences in gene expression profiles between BCB+ and BCB− oocytes [34]. Therefore, it is still largely unknown why mature metaphase II BCB− oocytes have low competency and how mtDNA deficiency contributes to oocyte quality, especially at the transcript level and whether the mitochondrial genome can influence the oocyte’s nuclear genome.

This study aimed to address two key questions using the pig as our model. First, we sought to determine the association between mtDNA deficiency and the transcription profiles of metaphase II oocytes. We undertook this utilising the most up to date version of the *Sus scrofa* reference genome and its annotations [35], and we included all the Ensembl annotated genes [36] to analyse single cell oocyte data [31]. Second, we sought to determine if the transcriptomic profiles during the developmental transition from the oocyte to the blastocyst would be affected following mtDNA supplementation. This was undertaken by employing a longitudinal analysis of differentially expressed genes [37,38]. Our analysis uncovered functional pathways affected in BCB− oocytes and altered the developmental gene expression in blastocysts as a result of mtDNA supplementation.

## 2. Results

### 2.1. mtDNA Deficient Oocytes Revealed Downregulation of OXPHOS Genes

mtDNA deficiency in *Sus scrofa* oocytes correlates significantly with BCB staining [13,14]. We previously analysed RNAseq data sets comprising BCB positive (BCB+) and negative (BCB−) oocytes (Methods Section 4.1 and Figure 1A) to address the effect of mtDNA deficiency on global oocyte transcriptome profiles [31] using the *Sus scrofa* reference genome Sscrofa10.2 (Accession No. GCF_000003025.5, released on 7 September 2011) [32]. However, more than half of the differentially expressed genes (DEGs) did not have proper annotation with gene symbols, and the results did not include read counts for the mtDNA encoded genes [31]. We reanalysed these BCB+ and BCB− oocyte data sets (Table S1) using the latest reference genome Sscrofa11.1 Accession No. GCF_000003025.6 [35] and Ensembl annotation release 105 [36] to determine if there would be greater clarity about the genes affected and the associated functional pathways and networks.

Single oocyte RNAseq data contained high numbers of sample replicates, i.e., 15 BCB+ and 14 BCB− oocytes (Methods Section 4.1 and Section 4.4). In all, 10 to 30 million reads per sample were analysed, and the levels of read mapping and assignment of annotation (Table S1) were comparable with previous analyses [39,40]. Principal component analysis (PCA) revealed high consistency for oocyte RNAseq profiles relative to blastocyst RNAseq data, as previously shown [19] (Figure S1A). We found that the RNAseq profiles of the oocytes were significantly affected by ovary source (Figure S1B, left panel) suggesting a significant effect of genetic background on each oocyte’s transcriptome profile. Therefore, ovary source was included in the data analysis, and the effect of batch was removed (Figure S1B, right panel).

**Figure 2:**
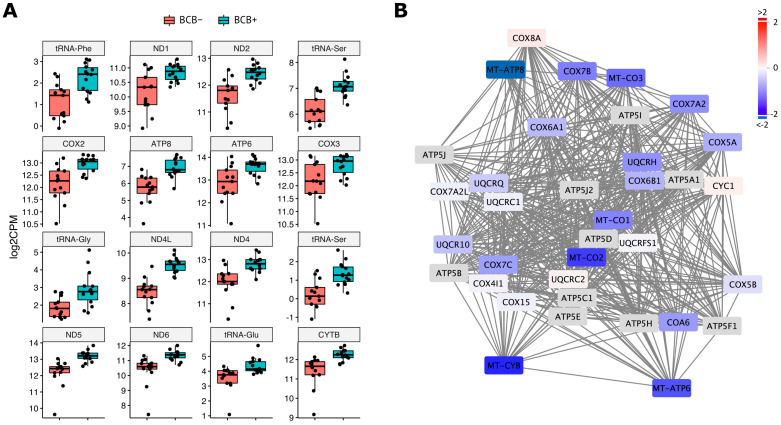
The expression of the mtDNA encoded and OXPHOS related genes in *Sus scrofa* oocytes. (**A**) mtDNA encoded genes and RNAs were differentially expressed between BCB+ and BCB− at significance (FDR < 0.05) and presented by jittered-boxplot. The results of individual transcripts are presented by positional order in the mitochondrial genome from top left to bottom right. Levels of expression were plotted on the y-axis as log2CPM value. (**B**) Potential protein interactions of identified DEGs involved in OXPHOS electron transport chain complexes III and IV and ATP synthase. The entire OXPHOS related protein interaction network is shown in Figure S2. Levels of differential gene expression between BCB+ and BCB− oocytes are shown as fold change by colour scale. Red and blue represent up- and down-regulation in BCB− oocytes. Genes in grey box have no DEG data due to low or no expression.

An analysis of the transcriptome profiles between BCB+ and BCB− oocytes identified 402 DEGs at a significance of FDR < 0.05 (Table 1 and Table S2). Amongst those, mtDNA encoded genes were abundant, including 11 protein coding genes and five tRNAs that were all significantly downregulated in BCB− oocytes (Figure 2A). In addition, *cytochrome c oxidase I* (*COXI*) and *NADH dehydrogenase 3* (*ND3*) also showed downregulation at an FDR < 0.1 indicating all protein coding genes of mtDNA were downregulated in BCB− oocytes. This is consistent with the lower levels of mtDNA copy number found in BCB− oocytes [13] resulting in reduced transcriptional activity for the mitochondrial genome. Gene ontology (GO) enrichment analysis of the whole DEG data sets identified genes involved in OXPHOS including ATP synthesis coupled electron transport, oxidative phosphorylation, and mitochondrial ATP synthesis, which were enriched (Table S3). Therefore, mtDNA deficiency in BCB− oocytes not only affected mtDNA encoded gene transcription, but also resulted in the downregulation of the majority of OXPHOS genes, which includes genes encoded by the nucleus (Figure 2B and Figure S2).

### 2.2. RNA Metabolism and Meiosis/Mitosis Related Gene Expression Were Affected in BCB− Oocytes

**Figure 3:**
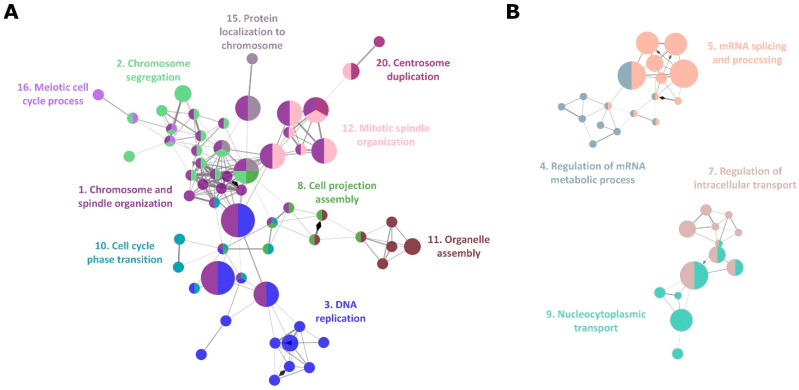
Functional pathway and gene network for DEGs between BCB+ and BCB− oocytes. (**A**) Enrichment of genes associated with meiosis and mitosis related functional pathways in GO biological process, identified by ClueGO. (**B**) Enrichment of genes associated with mRNA metabolism and intracellular transport related functional pathways. GO biological process terms in the same group are coloured and presented with their representative group names. The number associated with each group name corresponds to the GO group number in Table S4.

DEG analysis also identified 308 protein coding nuclear genes as well as 74 non-coding transcripts (Table S2). Ten out of the top 30 DEGs were small nucleolar RNAs (snoRNA) (Table 1) with a total of 56 snoRNA found to be downregulated in BCB− oocytes (Table 2). The primary function of snoRNA is the chemical modification of RNAs, mainly ribosomal RNA, transfer RNA, and small RNAs [41,42]. A recent study identified a noncanonical function for snoRNA, such as the regulation of chromatin structure and mRNA splicing. Consequently, the downregulation of a large subset of snoRNAs could affect RNA metabolism. Indeed, genes associated with mRNA processing and metabolism were enriched in the DEGs (Table S2), and therefore, corresponding GO and REACTOME terms (Tables S4 and S5) were identified as functional network groups (Figure 3B).

The most affected functional network in BCB− oocytes was the meiosis and mitosis cell cycle process (Figure 3A). A total of ten groups of GO terms associated with oocyte maturation and processes of cell division were found to be enriched in DEGs (Table S4). This suggests that poor oocyte quality is associated with the mis-regulation of genes required for the completion of metaphase II and following the first zygotic cell division after fertilization with the male gamete. Overall, mtDNA deficiency in BCB− oocytes affects the transcription of genes encoded by the nuclear genome that are essential for oocyte development and maturation and RNA metabolism, which, in turn, could result in the oocyte being defective.

### 2.3. The Use of mtDNA Supplementation in Oocytes Influences the Expression of Genes Involved in Development and Differentiation in Blastocysts

**Figure 4:**
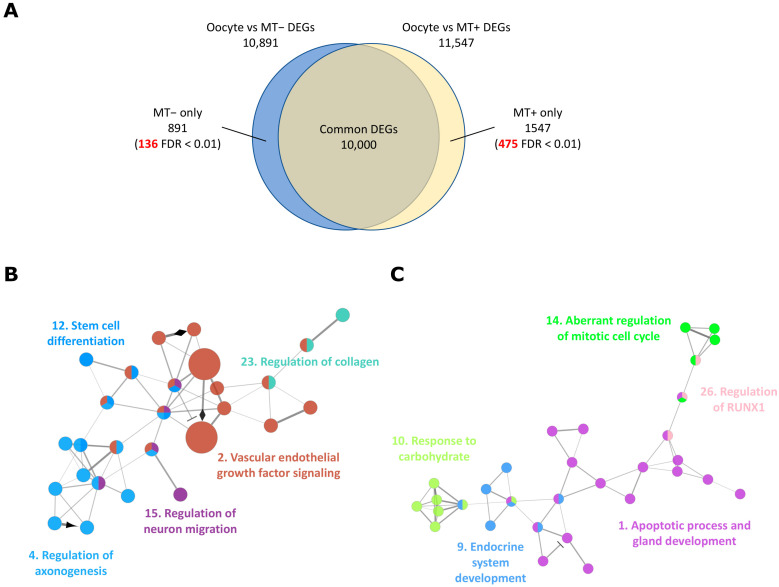
Functional pathways enriched in the DEGs were only found in oocyte to MT supplemented blastocyst transition, identified by longitudinal analysis. (**A**) Venn diagram representing common and unique DEGs from the two oocyte-blastocyst transition processes shown in Figure 1B. Numbers indicated in red are highly significant DEGs with FDR < 0.01 within unique DEGs from one of the transition processes. (**B**) Network associated with differentiation and development to blastocyst. (**C**) Network associated with gland and pancreas development. GO biological process and REACTOME terms in the same group are coloured and presented with their representative group name. The number for each group name corresponds to the GO and REACTOME group number in Table S9.

Transcriptional profiles change greatly during the developmental transition from a quiescent state in the oocyte to an active state in the developing embryo [43,44]. We have previously demonstrated the use of longitudinal analysis by assessing differentially methylated regions of the *Sus scrofa* genome from oocytes to blastocysts, which provided an additional layer of information and insights into the effect of mitochondrial supplementation [19]. We utilized this approach to analyse our RNAseq data sets of oocytes and blastocysts generated by intracytoplasmic sperm injection accompanied by (MT+) or without mtDNA (MT−) supplementation (Figure 1B). We hypothesized that the longitudinal analysis [37,38] would highlight the effect of mtDNA supplementation on blastocyst generation. To do this, DEGs between oocytes and blastocysts without mtDNA supplementation and between oocytes and blastocysts with supplementation were identified independently (Methods Section 4.1, Section 4.2, Section 4.3 and Section 4.4 and Figure 1B). We then compared the two lists of DEGs to determine if there were any unique DEGs in either of the developmental transition processes (Figure 1B and Figure 4A).

Approximately 11,000 genes were differentially expressed between the oocyte stage and blastocyst stage, of which 90% of DEGs (10,000 genes) were common irrespective of whether mtDNA supplementation had taken place (Figure 4A). In all, 891 genes were identified from the list of DEGs for the transition from oocyte to blastocyst generated without additional mtDNA. Of those genes, 136 had an FDR < 0.01 (Table 3 and Table S6). However, this subset of genes was not significantly different between the transition from oocytes to blastocysts that were supplemented (Figure S3A). Similarly, 1547 genes were only identified in the DEGs from the transition from oocyte to blastocysts supplemented with mtDNA. Of these, 475 genes had an FDR < 0.01, and most were highly expressed in the blastocysts (Figure S3B and Table S7). Some of these genes have been previously identified with a direct comparison between supplemented and non-supplemented blastocysts through RNAseq. For example, these include several tRNAs, *sestrin 1* (*SESN1*), *glycerophosphocholine phosphodiesterase 1* (GPCPD1), and *NIMA related kinase 2* (*NEK2*) [19]. Many others were successfully uncovered through the longitudinal analysis, complementing the direct comparison method, and further addressing the effect of mtDNA supplementation in a developmental context.

The non-supplemented blastocysts showed slightly less transcriptional change during transition and the relatively lower number of DEGs were unique to this transition (Figure 4A). Only four associated GO and REACTOME terms were enriched (Table S8). On the other hand, mtDNA supplementation of oocytes induced greater levels of gene activation in blastocysts, mainly nuclear-encoded protein coding genes (Table S7) and 211 GO and REACTOME terms, which consisted of 53 functional annotation groups that were enriched in this subset of DEGs (Table S9). Genes involved in cell differentiation and various tissue development, including the pancreas, glands, and neurons, were abundant and uniquely regulated in the process of transition to blastocyst development following supplementation (Figure 4B,C). Several of these genes, such as *BCL2 antagonist/killer 1* (*BAK1*), *neuregulin 1* (*NRG1*), *platelet derived growth factor receptor alpha* (*PDGFRA*), and *hepatocyte nuclear factor 4 alpha* (*HNF4A*), have critical roles in developmental processes [45,46,47,48]. The lack of activation of these genes in the non-supplemented blastocysts might have detrimental effects on early embryo development or be indictive of the premature expression of genes of early differentiation. Overall, the results of the longitudinal DEG analysis highlighted a novel aspect of the oocyte-to-blastocyst transition process.

### 2.4. Effect of mtDNA Supplementation on Parent of Origin Gene Expression Patterns

We have previously shown that mtDNA supplementation was associated with only minor differences in the DNA methylation of imprinted genes in *Sus scrofa*, and no differences were found in the levels of imprinted gene expression between supplemented and non-supplemented blastocysts [19]. However, it is still unknown if they showed parent of origin imprinted gene expression patterns. Therefore, we investigated the allele-specific expression of imprinted genes and neighbouring genes by analysing the frequency of single nucleotide polymorphisms (SNP) [49]. Since we used commercial pig sperm and oocytes collected from ovaries processed in a local Australian abattoir, it was not feasible to track parental genotypes from available resources. Therefore, we analysed SNPs in the transcripts, which reflect the expression of both parental alleles. If only one of the parental alleles is dominantly active for transcription as in imprinted genes, we would expect to find no or low numbers of SNPs in the transcript.

**Figure 5:**
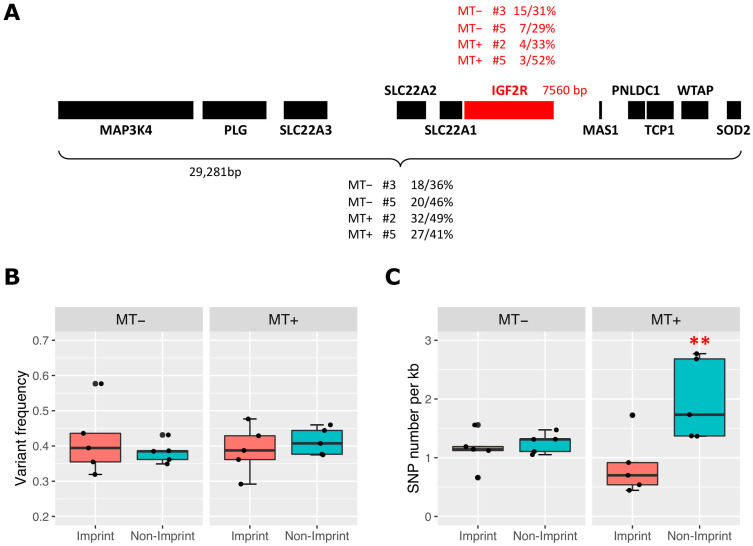
Bi-allelic expression of genes on imprinted loci from blastocysts with (MT+) or without (MT−) mtDNA supplementation. (**A**) Schematic representation of the *IGF2R* imprinting locus showing levels of bi-allelic transcripts. Boxes represent imprinted (red) and non-imprinted (black) genes on the locus. Identified SNP number/frequency of variants (%) in representative blastocyst RNAseq data are shown above (imprinted gene, red) or below (sum of non-imprinted genes, black) the boxes. Transcript size of *IGF2R* and sum of transcript size for non-imprinted genes at the locus are also presented. (**B**) Level of variant frequency of SNPs in imprinted and non-imprinted genes is shown by jittered-boxplot for MT− and MT+ blastocyst RNAseq. (**C**) Identified SNP frequency normalized by size of transcript (per kb) in imprinted and non-imprinted genes as shown in (**B**). Significant difference (**, *p* < 0.05) identified by ANOVA and Tukey test is indicated by asterisks.

The method requires genetic variations between the two parental pigs. Average SNP frequency in the human genome is about one in every 300 base pairs with a minor allele frequency greater than 1% [50,51,52], and an NGS analysis of four swine breeds using reduced representation genomic libraries identified 372K SNPs [53]. Therefore, it seems feasible that the allele-specific expression could be analysed by SNP identification with the depth of our blastocyst RNAseq data. We set up detection criteria for the number of reads per transcript, depth of sequence reads, number of SNPs per transcript, and level of a variant, as described in the Methods, to analyse allele-specific expression of genes at the imprinting loci. For example, *insulin like growth factor 2 receptor* (*IGF2R*) on *Sus scrofa* chromosome 1, one of the most characterized imprinted genes in human and mouse genomes [54,55,56], encodes 7560 bp transcripts (Accession no NM_001244473). In blastocyst sample MT+ #5, derived through supplementation, its RNAseq data identified three SNPs with a mean variant frequency of 52% (Figure 5A). In the same data, 27 SNPs were identified in a total transcript length of 29,281 bp containing 10 neighbouring genes. SNP frequency, calculated by SNP number divided by transcript length (kb), was used as an indicator of mono-allelic or bi-allelic expression at low and high frequency, respectively. In the RNAseq data set MT+ #5 (mtDNA supplemented blastocyst), the SNP frequency for *IGF2R* was 0.40 SNP per kb transcript (SNP/kb) whilst there was a 0.92 SNP/kb frequency for its neighbouring genes. This suggests that *IGF2R* was biased to mono-allelic expression as expected for imprinted genes. Similarly, in RNAseq data set MT− #3 (non-supplemented), the SNP frequency for *IGF2R* was 1.98 SNP/kb and 0.61 SNP/kb for its neighbouring genes. The even higher SNP frequency for *IGF2R* than its neighbouring genes suggests that both parental alleles were actively transcribed for this gene in this blastocyst.

It is possible that a low or high SNP frequency may simply reflect the genetic variation of parents for specific genes and genomic regions. To minimize distribution bias of the SNPs in specific genomic regions, which affects bi-allelic expression, we analysed 10 imprinted genes and 73 non-imprinted neighbouring genes at six imprinting loci across four chromosomes. Total transcript lengths of 57,219 bp and 273,582 bp were analysed for imprinted genes and non-imprinted neighbouring genes, respectively, and we normalized SNP data by length for comparison. Table S10 summarises the SNP analysis results, and the sex of each blastocyst was included in the model of statistical analysis. For genes with bi-allelic expression, about 50% variant frequency is expected, and we found ~40% variant frequency in non-imprinted genes from both supplemented and non-supplemented blastocysts (Figure 5B). Variant frequency in imprinted genes was similar, and this was understandable as we filtered and kept SNPs with the signature of bi-allelic expression. As mentioned above, it would be SNP frequency (SNP number per transcript length) that provides the status of allelic expression. In MT− blastocysts, there were no differences in SNP frequency between imprinted and non-imprinted genes (Figure 5C, left panel). This was interesting as the mono-allelic expression of imprinted genes is expected to contribute to a lower SNP frequency more than non-imprinted genes. Indeed, supplemented blastocysts showed a significantly lower SNP frequency in imprinted genes than non-imprinted genes (Figure 5C, right panel). As we analysed over 330 kb total transcript length for 83 genes, these were unlikely to be caused by specific genotype combinations of specific genes in non-supplemented blastocysts. However, this might suggest that blastocysts generated without mtDNA supplementation have delayed or disturbed imprinted gene expression or partial inactivation of one of the parental genomes for regular bi-allelic expression, or a combination of both.

## 3. Discussion

In this study, we have analysed RNAseq data sets from *Sus scrofa* oocytes and blastocysts generated by injecting sperm into a mature oocyte with or without mtDNA supplementation. RNAseq data were analysed globally using tagwise dispersion estimation and Generalized Linear Models (Methods Section 4.4) which provide unbiased estimates of the gene-specific dispersions. This approach is suitable for replicates with high variation, such as blastocyst RNAseq data. We thought validation by other tests, such as qRT-PCR, would be difficult for ultra-low quantity materials considering the associated technical and handling errors, selection of appropriate controls and detection thresholds, and variations amongst samples. The use of the most up-to-date *Sus scrofa* reference genome sequence and associated annotations identified differentially expressed genes in BCB− oocytes, including a large subset of snoRNAs as well as genes associated with meiotic and mitotic cell cycle processes, that were not identified in our previous analysis [31]. Furthermore, using a longitudinal transcriptome analysis approach [37,38] likely provided a greater range of DEGs and revealed activation of subsets of developmental genes in mtDNA supplemented blastocysts. This approach circumvented the issue of fewer genes being identified as a result of the direct comparison between MT+ and MT− blastocysts due to the high FDR values associated with high sample variations [19].

### 3.1. mtDNA Deficiency in BCB− Oocytes Associates with Downregulation of OXPHOS Genes

The developmental competence of oocytes has been assessed with BCB labelling in various mammals with BCB− oocytes reportedly associated with a lower fertilisation potential, lower frequency of development to blastocyst, lower levels of mitochondrial activity, changes in expression of genes involved in apoptosis, and lower levels of mtDNA copy number [21,24,25,28,30,57]. Our results provide further evidence that BCB− oocytes have highly distinct transcription profiles compared to BCB+ oocytes (Table 1 and Table S2 and Figure 2). A total of 16 mtDNA encoded genes, including 11 protein coding and 5 t-RNAs, were significantly downregulated in BCB− oocytes with two mtDNA protein coding genes, *COXI* and *ND3*, also downregulated but to levels of lower significance (FDR < 0.1). Since mtDNA copy number in pig BCB− metaphase II oocytes is ~3-fold lower than in BCB+ oocytes [13] and that there are strong associations in other species, such as the human [8], pig [58], and mouse [59], with mtDNA copy number and fertilisation outcome, this was not particularly surprising. However, it confirmed low levels of expression for all of the mtDNA-encoded genes that are associated with OXPHOS and that the majority of nuclear-encoded genes associated with OXPHOS were also downregulated in BCB− oocytes (Table 1 and Table S2 and Figure 2 and Figure S2). On the other hand, we did not find differences in the expression of genes involved in mtDNA replication, such as *DNA polymerase gamma* (*POLG*), *mitochondrial transcription factor A* (*TFAM*), and *nuclear respiratory factor 1* (*NRF1*), which have been previously reported to be differentially expressed in BCB− oocytes [30,57], although it should be pointed out that the previous studies analysed these genes in isolation with qRT-PCR and not as part of large data sets. Overall, the association between BCB− oocytes and the downregulation of OXPHOS-related genes are consistent with the previous observations that BCB− oocytes have lower levels of mtDNA copy number and mitochondrial activity [13,21,24,25].

### 3.2. Downregulation of RNA Metabolism and Meiotic and Mitotic Cell Cycle Process Genes in mtDNA Deficient Oocytes

One of the most striking differences between the transcriptome profiles for BCB+ and BCB− oocytes was the downregulation of a large subset of snoRNAs (Table 2). snoRNAs play a central role in ribosome biogenesis, guiding the sequence-specific chemical modification of pre-rRNA and its processing [41,42,60]. snoRNAs are components of ribonucleoprotein complexes and are divided into two main classes based on highly conserved sequences, namely box C/D and box H/ACA motifs. In addition, some snoRNAs of both classes are localized in Cajal bodies, known as small Cajal body-associated RNAs (scaRNA). We determined these three types of snoRNAs were downregulated in BCB− oocytes (Table 2). The main functions of canonical snoRNAs and scaRNAs are their catalysation of 2’-O-ribose methylation (box C/D) and guidance of the pseudouridylation (box H/ACA) of rRNA and small nuclear RNAs, respectively. Subsets of snoRNAs have noncanonical functions, including the regulation of splicing, mediators of oxidative stress, and regulation of chromatin structure [41,61]. In vertebrates, snoRNAs are encoded in the introns of host genes, protein coding mRNA or long noncoding RNA, and released from the host gene precursor RNAs by splicing. After the splicing and production of snoRNAs, host gene RNAs are directed to degradation via nonsense-mediated RNA decay (NMD) [62], and NMD genes were significantly affected in BCB− oocytes (REACTOME Group 3 in Table S5). Although there are known functions for snoRNAs in RNA biogenesis and metabolism, as mentioned above, the functional annotations of snoRNA genes (*SNORD* and *SNORA*) themselves were not readily available for the GO and REACTOME enrichment analysis [63,64]. Therefore, snoRNAs did not contribute to the enrichment analysis results, and no *SNORD* and *SNORA* genes were found in Tables S3–S5 (in ‘Associated Genes’ columns). Despite this, the RNA metabolic process, catabolic process, and splicing and processing were still found to be significantly enriched (Tables S4 and S5 and Figure 3B) indicating that RNA metabolism is largely affected in BCB− oocytes.

The most affected functional network in BCB− oocytes was meiotic and mitotic cell cycle processes, including chromosome segregation, spindle organization, DNA replication, centrosome duplication, and cell cycle phase transition (Figure 3A and Tables S4 and S5). After maturation in the ovary or in medium, oocyte development is arrested at the metaphase II stage prior to fertilisation [65]. Fertilisation triggers the completion of metaphase II by segregating half of the sister chromatids into the second polar body and forming a female haploid pronucleus to fuse with the male pronucleus. The zygote then assembles the first mitotic spindle for the first cell division. Genes involved in these cell cycle processes were differentially expressed in BCB− oocytes and were mostly downregulated (Tables S2 and S5). It is reasonable to assume that the lack of these critical gene transcripts in readiness for the completion of meiosis II and following the first mitotic division could cause significant developmental defects at fertilisation and during embryogenesis. These might include failed chromosomal segregation and subsequent aneuploidies that lead to embryonic arrest or spontaneous abortion [66,67]. The downregulation of RNA metabolism and processing is likely to be part of the cause of the under-representation of those critical cell cycle gene transcripts.

### 3.3. mtDNA Supplementation Maintains Expression of Genes Necessary for Embryogenesis

Longitudinal DEG analysis identified subsets of genes that are differentially expressed in mtDNA supplemented-derived blastocysts, developed from quiescent state oocytes (Table S7). Those genes were mostly upregulated in the supplemented blastocysts and involved in various developmental processes (Figure 4 and Table S9). These include *BAK1* which functions as a pro-apoptotic regulator involved in a wide variety of cellular activities and shaping tissue and organ morphologies via apoptosis [48,68,69]. In addition, PDGFRA is a receptor kinase binding to PDGF that stimulates the growth and migration of vascular smooth muscle cells, fibroblasts, and glial cells [46,70,71]. On the other hand, deletion of *PDGFRA* induces embryonic lethality and causes the defective development of many endoderm- and mesoderm-derived structures [72,73]. Zinc finger protein 792 (ZNF792) is a paralogue of ZNF304, which recruits repressive cofactors and induces DNA hypermethylation and transcriptional silencing [74]. HNF4A is a nuclear transcription factor that regulates the expression of several hepatic genes for the development of the liver, kidney, and intestines. Mutations in *HNF4A* are associated with a monogenic form of type 2 diabetes and the loss of function in mice, causing severe hepatomegaly and steatosis and resulting in premature death [45,75]. Moreover, NRG1 is a trophic factor that contains an epidermal growth factor (EGF)-like domain and NRG1 signalling is required for embryonic development. The loss of function of NRG1 can cause homozygous lethality and behavioural abnormalities in heterozygous mutant mice [47,76] whilst the addition of NRG1 to the oocyte in vitro maturation media results in improved embryo development [58]. These genes were activated in the supplemented blastocyst during the oocyte-to-blastocyst transition process, but not significantly upregulated in the non-supplemented blastocysts. Since mtDNA supplementation induces mtDNA replication in early embryogenesis and improves embryo quality [13], this likely enhances developmental gene expression during progression to the blastocyst stage.

Communication between the nuclear and mitochondrial genomes is important for effective cellular function. It is well-documented that ATP generated through OXPHOS requires the replication of the mitochondrial genome to have taken place [77] and that mtDNA copy is not uniform across all cell types but rather reflects the requirements of each cell type for OXPHOS-derived ATP [78]. Indeed, throughout development, there are changes in mtDNA copy that reflect the metabolic requirements of the developing embryo and foetus. For example, undifferentiated (pluripotent) cells have a requirement for low levels of OXPHOS-derived ATP [79], as is the case for tumour-initiating cells [80] whilst heart cells and neurons have high levels of the mtDNA copy number to support their complex functions [81]. A failure to mediate the interactions between the two genomes will inhibit key developmental processes, such as differentiation, and result in cellular arrest. However, the modulation of one genome can impact the other. For example, the use of DNA demethylation agents on tumour-initiating cells can result in increased differentiation potential and increased mtDNA copy number that take place in a synchronous fashion [82]. Likewise, the short-term depletion of mtDNA copy number can reset the differentiation potential of tumour-initiating cells whilst longer term depletion can inhibit tumorigenesis with both outcomes exhibiting differences in nuclear gene expression [80]. mtDNA deficiency in oocytes likely provides another example of how one genome is asynchronous with the other. Indeed, BCB− oocytes are indicative of poor developmental competence where nuclear and cytoplasmic maturation are not in synchrony. As a result, these oocytes frequently either fail to fertilise or arrest during preimplantation development [14,30], but they can be rescued through mtDNA supplementation [13]. Developmental competence can also be rescued through the addition of agents like NRG1 [58] or the glycoside mogroside V [83] to the oocyte maturation media, which impact the mtDNA copy number and enhance embryo development.

### 3.4. mtDNA Supplementation Might Improve Imprinted Gene Expression

Accumulated evidence suggests that assisted reproductive technologies, for example, in vitro embryo culture, in vitro fertilisation, and intracytoplasmic sperm injection affect imprinted gene methylation and expression compared to the children from spontaneous conception [84,85,86,87]. Embryos generated by in vitro fertilisation and cultured in medium can either show the bi-allelic expression of *H19* or limited paternal expression [85,88]. Moreover, differential DNA methylation and expression of *IGF2*/*H19* and *IGF2R* in placenta and cord blood have been reported for in vitro conceived children [89]. Furthermore, altered expression of *H19* and *Pleckstrin homology-like domain family A member 2* (*PHLDA2*) has been observed in human placentas following in vitro fertilisation and intracytoplasmic sperm injection, but this was not due to the loss of the pattern of imprinted gene expression [90]. Our previous study identified differentially methylated regions throughout the nuclear genome between blastocysts derived through either mtDNA supplementation or without, but they showed only minor differences in DNA methylation in imprinting control regions (ICRs) [19]. Differential DNA methylation patterns of ICRs, inherited from paternal and maternal genomes, are essential for imprinted gene expression and maintained in somatic cell lineages [55]. Although there was no difference in the level of imprinted gene expression associated with mtDNA supplementation [19], we observed a potential delay or disturbance of the establishment of the patterns of parental allele-specific imprinted gene expression in the non-supplemented blastocysts (Figure 5 and Table S10). We thought the effect of blastocyst sample variation on the imprinted gene expression pattern was minimal as DNA methylation of ICRs was already established prior to fertilisation and maintained in the blastocysts [19]. Blastocysts derived from mtDNA supplementation showed less bi-allelic expression in imprinted genes and higher levels in neighbouring non-imprinted genes, suggesting an improved establishment of parental allele-specific expression patterns. Combining mtDNA supplementation with specific embryo culture conditions [91] might be a potential approach to improve gene expression profiles so that they more closely resemble the profiles of naturally conceived embryos. However, this would require a full understanding of how the mtDNA supplementation approach enhances embryo development.

We have shown that mtDNA deficiency in *Sus scrofa* oocytes significantly affects the expression of genes involved in RNA metabolism and processing. Furthermore, the downregulation of genes associated with meiotic and mitotic cell cycle processes are likely to be one of the direct causes of poor oocyte quality and poor fertilisation outcome. However, further understanding of how mtDNA deficiency influences nuclear gene expression with respect to communication between the two genomes needs to be established. We also showed that the mtDNA supplementation of oocytes in combination with sperm injection improves the maintenance of developmental gene expression and patterns of parental allele-specific imprinted gene expression in blastocysts. These results further support the concept that the addition of extra copies of mtDNA has a significant impact on the overall gene expression profiles during oocyte-to-blastocyst transition and contribute to improved fertilisation and preimplantation development outcomes.

## 4. Materials and Methods

### 4.1. Oocyte Collection and BCB Staining

Cumulus–oocyte complexes (COCs) were collected from gilt ovaries obtained from a local abattoir and cultured as previously described [19,31]. Briefly, porcine ovaries were transported to the laboratory in warm 0.9% NaCl solution. The COCs were then aspirated from follicles with diameters of between 3 and 6 mm using an 18 G needle. The COCs were then washed 3 times in handling media (25 mM Hepes–TCM199, Gibco^®^, Waltham, MA, USA) supplemented with 10% sow follicular fluid (SFF). The COCs were incubated in 12 μM BCB in in vitro maturation (IVM) medium for selection as described in [13], and BCB+ and BCB− COCs were then washed and cultured separately for 42–44 h in pre-equilibrated IVM medium. To collect metaphase II (MII) oocytes, expanded COCs following IVM were briefly treated with 0.1% (0.5mg/mL) of hyaluronidase and gentle pipetting. The resultant denuded oocytes were washed using a narrow glass pipette to completely remove all cumulus cells (Figure 1A). Cohorts of single MII oocytes were collected and stored at −80 °C prior to RNA extraction [31].

### 4.2. Generation of Blastocysts by Intracytoplasmic Sperm Injection (ICSI) with (MT+) or without (MT−) mtDNA Supplementation

Expanded blastocyst stage *Sus scrofa* embryos were generated from in vitro matured oocytes by ICSI; and mitochondrial supplementation in combination with ICSI using third-party oocytes (heterologous) or sister oocytes (autologous) as the source of the mitochondrial isolate as previously described in [13,19]. Briefly, 15 oocytes were homogenised in mitochondrial isolation buffer (20 mM Hepes pH 7.6, 220 mM mannitol, 70 mM sucrose, 1 mM EDTA) containing 2 mg/mL BSA by a drill-fitted Teflon pestle. The homogenate was centrifuged at 800× *g* for 10 min at 4 °C to remove cell debris, and then, supernatants were centrifuged at 10,000× *g* for 20 min at 4 °C to pellet the mitochondrial fraction. Following two washing steps, the mitochondrial pellet was resuspended in 5 μL of mitochondrial buffer. For ICSI with mitochondrial supplementation, 3 pl of mitochondrial isolate along with a single sperm was injected into the oocyte as described [13]. Single embryos were stored in 0.2 mL PCR tubes in a −80 °C freezer prior to RNA extraction.

### 4.3. RNA Extraction, RNAseq Library Preparation, and Sequencing

Total RNA was extracted from single oocytes or single expanded blastocysts using the PicoPure RNA isolation kit (Thermo Fisher Scientific, Waltham, MA, USA), according to the manufacturer’s instructions. The quality of total RNA was assessed using High Sensitivity RNA ScreenTape (Agilent Technologies, Santa Clara, CA, USA). Next generation sequencing (NGS) libraries were prepared using Ovation^®^ RNA-Seq system V2 and Ovation^®^ Ultralow System V2 (NuGEN Technologies, Männedorf, Switzerland) for single MII oocytes [31] and the Trio RNA-Seq Library Preparation Kit (Tecan Group Ltd., Männedorf, Switzerland) for single blastocysts [19]. NGS libraries were sequenced using 100 bp paired-end sequencing chemistry.

### 4.4. RNAseq Data Analysis and Differentially Expressed Gene (DEG) Identification

Single oocyte (NCBI Sequence Read Archive IDs: SRR6451030—SRR6451058) and blastocyst (SRR16706437—SRR16706452) RNAseq data from our previous studies [19,31] were used. Oocyte (14 BCB− oocytes and 15 BCB+ oocytes) and blastocyst (5 MT− and 5 MT+) RNAseq sample metadata can be found in Table S1. RNAseq raw fastq files were quality checked with ‘*fastqc*’ (version 0.11.9) [92], and trimming of adapters and quality filtering were then performed with ‘*fastp*’ (version 0.20.1) [93] with options: *--detect_adapter_for_pe*, *-q 20*, *--length_required 30.* Trimmed and quality filtered paired-end reads were aligned to the *Sus scrofa* reference genome sequence Sscrofa11.1 accession No. GCF_000003025.6 [35] and Ensembl annotation release 105 [36] using ‘*STAR*’ (version 2.7) [94] with default parameters. Gene expression was quantified by counting the number of reads aligned to each Ensembl gene model using ‘*featureCounts*’ (version 1.5.2) [95], and output results were assessed for mapping quality by MultiQC version 1.9 [96]. Summary statistics for the RNAseq data are shown in Table S1. The Trimmed Mean of M-values (TMM) normalisation method from *edgeR* was applied to normalise read counts according to library size differences between samples. PCA was performed to visualise the summary of gene expression profiles for all RNAseq data. We found that the source of the ovary affected gene expression profiles identified by PCA; therefore, this was included in the linear mixed model as a covariate and batch effect was successfully corrected in the expression data.

For comparisons between BCB+ and BCB− oocytes, genes with low expression were filtered out prior to DEG analysis, keeping genes with at least 1 count per million (CPM) reads in more than eight samples. Data were analysed by using generalized linear models ‘*glmfit*’ and ‘*glmLRT*’ in *edgeR* version 3.32.0 [97] in R version 4.0.3 with the source of ovary as a covariate factor in the model. Genes were considered differentially expressed if their FDR (false discovery rate) or adjusted *p*-value was <0.05 (Table S2). Similarly for longitudinal DEG analysis, DEG analysis was carried out on RNAseq data from BCB+ oocytes and blastocysts generated with (MT+) or without (MT−) mtDNA supplementation, independently (Figure 1B). Then, two subsets of DEGs were compared to identify unique DEGs for the transition from oocyte to either MT− or MT+ blastocysts. In this analysis, genes with low expression were filtered out prior to DEG analysis, keeping genes with at least 1 count per million (CPM) reads in more than four samples. DEGs between BCB+ oocyte and MT− or MT+ blastocysts were identified using the limma-voom method (version 3.46.0) [98,99] to maintain consistency with the analysis tool used in a previous report [19]. An FDR threshold < 0.1 was used for significant DEGs between BCB+ oocytes and blastocysts with or without mtDNA supplementation and compared two subsets of DEGs for the efficient elimination of common DEGs. A Venn diagram was generated to show common and unique subsets of DEGs for each comparison (Figure 4A), and high confidence (FDR < 0.01) unique DEGs are listed in Tables S6 and S7. Results were visualized with jittered-boxplot, using ggplot2 [100].

### 4.5. Functional Pathway Enrichment and Gene Network Analysis

Functional pathway enrichment analyses for DEGs were performed using the *GSEA* v4.2.3 [64] and Enrichment Map [101] as described in [102]. Briefly, *Sus scrofa* Ensembl gene IDs were used to search for corresponding human orthologue gene symbols using the Ensembl BioMart database [103] and used for pathway enrichment analysis input. Annotation gene set file, Human_GO_AllPathways_with_GO_iea_May_25_2022_symbol.gmt from Bader lab gene sets collections (http://download.baderlab.org/EM_Genesets/current_release/, accessed on 8 February 2023), was used for *GSEA* analysis with a default FDR threshold of 0.25 (Table S3). The enrichment analysis results for GO biological process [104] and REACTOME pathway [105] were then visualized with the *Enrichment Map* app in Cytoscape [106]. We also used ClueGO [63] for pathway enrichment analysis with subsets of DEGs (Tables S4, S5, S8 and S9). For protein–protein interaction network analysis, the STRING [107] app in Cytoscape was used.

### 4.6. Bi-Allelic Expression Analysis of Genes in Imprinting Loci

Imprinted genes and neighbouring genes at the locus were analysed for the level of bi-allelic expression by identifying single nucleotide polymorphisms (SNP) in the mtDNA supplemented (MT+) and non-supplemented (MT−) blastocyst transcripts. *Sus scrofa* imprinted genes (https://www.geneimprint.com/site/genes-by-species.Sus+scrofa, accessed on 8 February 2023) with sufficient read depth were selected for analysis. We set a total of 5000 reads or more per imprinted gene transcript from ten blastocyst RNAseq data sets (MT−: *n* = 5 and MT+: *n* = 5) as a threshold to obtain sufficient read counts for SNP identification (Table S10). We analysed 10 imprinted genes and 73 non-imprinted neighbouring genes at six imprinting loci across four chromosomes. Imprinted genes and loci selected for investigation were: Chr1: *IGF2R* and *NDN* loci, Chr6: *OSBPL1A* and *DIRAS3* loci (including *SGIP1*), Chr9: *PEG10* locus (including *CASD1*, *PPP1R9A*), Chr14: *INPP5F* locus (including *TACC2*).

SNPs were identified and counted using ‘*samtools mpileup*’ for mapped reads pileup [108] and ‘*VarScan*’ version 2.3.8 [109] for variant sequence search. Imprinted gene locus sequences, including 2Mb upstream and downstream sequences of the *Sus scrofa* reference genome sequence Sscrofa11.1 accession No. GCF_000003025.6, were used for read pileup. Variant call by VarScan was made with the options: *--min-avg-qual 20 –min-var-freq 0.01 –min-reads2 10*, and the results were further filtered by keeping variant calls if there were >25 read counts per site and variant frequency was between 20–80%. Furthermore, if there were more than three SNPs per transcript, they were kept for further analysis as confident SNPs indicating bi-allelic expression status. SNPs less than three per transcript were discarded as they might be associated with technical issues, such as PCR and sequencing errors. SNP frequency was calculated by number of SNP counts per kb of transcript and mean variant frequency was also calculated (Table S10). ANOVA test was carried out to see statistical significance associated with blastocyst type (MT− and MT+) and imprinting status by including sex of blastocyst, date of sampling, and date of RNAseq library construction as covariate factors. Sex of each blastocyst was determined by assessing read counts of selected chromosome X- and Y-linked genes [110].

## Figures and Tables

**Table 1:** Differentially expressed genes between BCB+ and BCB− oocytes. Top 30 genes are short listed from Table S2.

gene_id ^a^	Symbol ^b^	Description	Chr/Scaffold ^c^	gene_biotype ^d^	logFC ^e^	logCPM ^f^	FDR ^g^
ENSSSCG00000018196	SNORD57	Small nucleolar RNA, C/D box 57 [Source:HGNC Symbol;Acc:HGNC:10207]	17	snoRNA	−1.607	3.130	8.85 × 10^−9^
ENSSSCG00000018086	ND4L	NADH dehydrogenase subunit 4L [Source:NCBI gene (formerly Entrezgene);Acc:808509]	MT	protein_coding	−1.056	9.173	2.91 × 10^−6^
ENSSSCG00000018076	tRNA-Ser	product=tRNA-Ser	MT	Mt_tRNA	−0.928	6.811	5.00 × 10^−6^
ENSSSCG00000018715	Y_RNA	Y RNA [Source:RFAM;Acc:RF00019]	9	Y_RNA	−1.681	4.741	7.51 × 10^−6^
ENSSSCG00000035954	ATAD5	ATPase family AAA domain containing 5 [Source:VGNC Symbol;Acc:VGNC:85601]	12	protein_coding	−0.490	8.050	1.13 × 10^−5^
ENSSSCG00000027476		Novel gene	17	snoRNA	−1.747	3.181	1.51 × 10^−5^
ENSSSCG00000019375	Y_RNA	Y RNA [Source:RFAM;Acc:RF00019]	9	Y_RNA	−1.958	5.446	1.63 × 10^−5^
ENSSSCG00000018080	ATP8	mitochondrially encoded ATP synthase membrane subunit 8 [Source:VGNC Symbol;Acc:VGNC:99789]	MT	protein_coding	−1.252	6.576	1.63 × 10^−5^
ENSSSCG00000038361		Novel gene	4	scaRNA	−1.400	3.834	3.28 × 10^−5^
ENSSSCG00000007427	ZSWIM3	zinc finger SWIM-type containing 3 [Source:VGNC Symbol;Acc:VGNC:95761]	17	protein_coding	0.392	8.218	3.37 × 10^−5^
ENSSSCG00000018864	SNORD89	Small nucleolar RNA, C/D box 89 [Source:HGNC Symbol;Acc:HGNC:32750]	3	snoRNA	−1.026	4.729	3.37 × 10^−5^
ENSSSCG00000001075	DEK	DEK proto-oncogene [Source:HGNC Symbol;Acc:HGNC:2768]	7	protein_coding	−0.412	8.286	7.78 × 10^−5^
ENSSSCG00000018402		Novel gene	6	snoRNA	−1.254	4.842	7.78 × 10^−5^
ENSSSCG00000018672	U5	U5 spliceosomal RNA [Source:RFAM;Acc:RF00020]	1	snRNA	−1.548	4.437	7.78 × 10^−5^
ENSSSCG00000022137		Novel gene	6	snoRNA	−1.445	2.871	8.33 × 10^−5^
ENSSSCG00000019644		Novel gene	9	snoRNA	−1.402	2.864	8.33 × 10^−5^
ENSSSCG00000014075	NSA2	NSA2 ribosome biosis factor [Source:VGNC Symbol;Acc:VGNC:100315]	2	protein_coding	−0.571	7.335	8.33 × 10^−5^
ENSSSCG00000000782	C12orf40	chromosome 12 open reading frame 40 [Source:HGNC Symbol;Acc:HGNC:26846]	5	protein_coding	−0.459	7.285	8.49 × 10^−5^
ENSSSCG00000016488	SSBP1	single stranded DNA binding protein 1 [Source:VGNC Symbol;Acc:VGNC:93479]	18	protein_coding	−0.616	5.067	1.03 × 10^−4^
ENSSSCG00000022479	UBR5	ubiquitin protein ligase E3 component n-recognin 5 [Source:VGNC Symbol;Acc:VGNC:94671]	4	protein_coding	0.356	7.585	1.55 × 10^−4^
ENSSSCG00000032111	RPL7	ribosomal protein L7 [Source:VGNC Symbol;Acc:VGNC:98850]	4	protein_coding	−0.412	6.888	1.70 × 10^−4^
ENSSSCG00000000863	SYCP3	synaptonemal complex protein 3 [Source:HGNC Symbol;Acc:HGNC:18130]	5	protein_coding	−0.484	5.853	1.70 × 10^−4^
ENSSSCG00000013249	CKAP5	cytoskeleton associated protein 5 [Source:VGNC Symbol;Acc:VGNC:86717]	2	protein_coding	0.509	7.481	1.79 × 10^−4^
ENSSSCG00000040136		NULL	12	snoRNA	−1.745	1.512	2.23 × 10^−4^
ENSSSCG00000030175	SNORD8	Small nucleolar RNA, C/D box 8 [Source:HGNC Symbol;Acc:HGNC:20159]	7	snoRNA	−1.117	5.160	2.27 × 10^−4^
ENSSSCG00000005201	UHRF2	ubiquitin like with PHD and ring finger domains 2 [Source:VGNC Symbol;Acc:VGNC:94692]	1	protein_coding	0.437	10.462	2.27 × 10^−4^
ENSSSCG00000025540	SNORD33	Small nucleolar RNA Z195/SNORD33/SNORD32 family [Source:RFAM;Acc:RF00133]	6	snoRNA	−1.147	3.188	2.67 × 10^−4^
ENSSSCG00000003820	USP1	ubiquitin specific peptidase 1 [Source:VGNC Symbol;Acc:VGNC:94742]	6	protein_coding	−0.384	8.692	3.01 × 10^−4^
ENSSSCG00000018456	SNORD49A	Small nucleolar RNA, C/D box 49A [Source:HGNC Symbol;Acc:HGNC:10189]	12	snoRNA	−1.348	3.024	3.01 × 10^−4^
ENSSSCG00000022145	RPF1	ribosome production factor 1 homolog [Source:VGNC Symbol;Acc:VGNC:92421]	6	protein_coding	−0.427	5.988	3.09 × 10^−4^

^a^, Ensembl (https://m.ensembl.org/index.html, accessed on 8 February 2023) gene ID. ^b^, Gene symbol. ^c^, Chromosome or scaffold number where gene is located. ^d,^ A gene or transcript classification. Mt_tRNA, mitochondrial genome encoded tRNA; protein_coding, protein coding genes; snoRNA, small nucleolar RNAs; Y_RNA, small non-coding RNAs that are components of the Ro60 ribonucleoprotein particle. ^e^, Log2 fold change relative to BCB+ oocytes. ^f^, Average expression levels, shown in TMM-normalised log2 count per million (CPM) sequence reads. ^g^, Significant difference in expression between BCB− and BCB+ oocytes by FDR test (*p* < 0.05).

**Table 2:** Small nucleolar RNA differentially expressed between BCB+ and BCB− *Sus scrofa* oocytes.

gene_id ^a^	Symbol ^b^	Description	Chr/Scaffold ^c^	Family ^d^	Target RNA ^e^	logFC ^f^	logCPM ^g^	FDR ^h^
ENSSSCG00000018196	SNORD57	Small nucleolar RNA, C/D box 57 [Source:HGNC Symbol;Acc:HGNC:10207]	17	C/D	18S rRNA	−1.607	3.130	8.85 × 10^−9^
ENSSSCG00000027476		NULL	17	na		−1.747	3.181	1.51 × 10^−5^
ENSSSCG00000038361		NULL	4	scaRNA		−1.400	3.834	3.28 × 10^−5^
ENSSSCG00000018864	SNORD89	Small nucleolar RNA, C/D box 89 [Source:HGNC Symbol;Acc:HGNC:32750]	3	C/D	unknown	−1.026	4.729	3.37 × 10^−5^
ENSSSCG00000018402		NULL	6	na		−1.254	4.842	7.78 × 10^−5^
ENSSSCG00000022137		NULL	6	na		−1.445	2.871	8.33 × 10^−5^
ENSSSCG00000019644		NULL	9	na		−1.402	2.864	8.33 × 10^−5^
ENSSSCG00000040136		NULL	12	na		−1.745	1.512	2.23 × 10^−4^
ENSSSCG00000030175	SNORD8	Small nucleolar RNA, C/D box 8 [Source:HGNC Symbol;Acc:HGNC:20159]	7	C/D	U6 snRNA	−1.117	5.160	2.27 × 10^−4^
ENSSSCG00000025540	SNORD33	Small nucleolar RNA Z195/SNORD33/SNORD32 family [Source:RFAM;Acc:RF00133]	6	C/D	18S rRNA	−1.147	3.188	2.67 × 10^−4^
ENSSSCG00000018456	SNORD49A	Small nucleolar RNA, C/D box 49A [Source:HGNC Symbol;Acc:HGNC:10189]	12	C/D	28S rRNA	−1.348	3.024	3.01 × 10^−4^
ENSSSCG00000028483	SNORD33	Small nucleolar RNA Z195/SNORD33/SNORD32 family [Source:RFAM;Acc:RF00133]	6	C/D	18S rRNA	−1.002	3.777	3.66 × 10^−4^
ENSSSCG00000040143	SNORA58B	Small nucleolar RNA, H/ACA box 58B [Source:HGNC Symbol;Acc:HGNC:52208]	4	H/ACA	28S rRNA	−1.716	1.360	4.32 × 10^−4^
ENSSSCG00000019590	SNORA27	small nucleolar RNA, H/ACA box 27 [Source:HGNC Symbol;Acc:HGNC:32617]	11	H/ACA	28S rRNA	−1.194	3.376	4.82 × 10^−4^
ENSSSCG00000033614		NULL	6	na		−2.000	0.984	5.45 × 10^−4^
ENSSSCG00000035503		NULL	AEMK02000697.1	na		−1.000	5.218	7.66 × 10^−4^
ENSSSCG00000019612	SNORD24	Small nucleolar RNA SNORD24 [Source:RFAM;Acc:RF00069]	9	C/D	28S rRNA	−1.331	2.750	8.09 × 10^−4^
ENSSSCG00000019338		NULL	4	na		−1.577	1.848	1.35 × 10^−3^
ENSSSCG00000020458		NULL	X	scaRNA		−1.668	2.903	1.60 × 10^−3^
ENSSSCG00000029549	SNORD22	Small nucleolar RNA SNORD22 [Source:RFAM;Acc:RF00099]	2	C/D	18S rRNA	−1.182	2.735	1.60 × 10^−3^
ENSSSCG00000019780	SNORD83	Small nucleolar RNA SNORD83 [Source:RFAM;Acc:RF00137]	7	C/D	unknown	−1.499	1.512	1.60 × 10^−3^
ENSSSCG00000028290	SNORD27	Small nucleolar RNA SNORD27 [Source:RFAM;Acc:RF00086]	2	C/D	18S rRNA	−1.383	1.616	3.12 × 10^−3^
ENSSSCG00000019046	SNORD110	Small nucleolar RNA, C/D box 110 [Source:HGNC Symbol;Acc:HGNC:32775]	17	C/D	18S rRNA	−2.087	0.945	4.35 × 10^−3^
ENSSSCG00000019944	SNORD97	Small nucleolar RNA, C/D box 97 [Source:HGNC Symbol;Acc:HGNC:32760]	2	C/D	unknown	−0.988	4.540	4.53 × 10^−3^
ENSSSCG00000024084		NULL	17	na		−1.510	1.048	4.59 × 10^−3^
ENSSSCG00000018197		NULL	6	na		−1.243	2.643	5.82 × 10^−3^
ENSSSCG00000025513	SNORD22	Small nucleolar RNA SNORD22 [Source:RFAM;Acc:RF00099]	2	C/D	18S rRNA	−1.071	3.251	8.62 × 10^−3^
ENSSSCG00000026212	SNORD29	Small nucleolar RNA SNORD29 [Source:RFAM;Acc:RF00070]	2	C/D	28S rRNA	−1.440	1.514	0.010
ENSSSCG00000037405	SNORD36	Small nucleolar RNA SNORD36 [Source:RFAM;Acc:RF00049]	1	C/D	18S rRNA	−1.031	2.604	0.011
ENSSSCG00000039700		NULL	6	na		−1.330	2.229	0.011
ENSSSCG00000032033	SNORD116	Small nucleolar RNA SNORD116 [Source:RFAM;Acc:RF00108]	AEMK02000602.1	C/D	unknown	−0.877	4.043	0.011
ENSSSCG00000019956	SNORD105B	Small nucleolar RNA, C/D box 105B [Source:HGNC Symbol;Acc:HGNC:33572]	2	C/D	18S rRNA	−1.660	0.451	0.014
ENSSSCG00000020372		NULL	5	scaRNA		−1.262	2.978	0.015
ENSSSCG00000019894	SNORA62	Small nucleolar RNA SNORA62/SNORA6 family [Source:RFAM;Acc:RF00091]	13	H/ACA	28S rRNA	−1.210	2.391	0.015
ENSSSCG00000030032		NULL	13	na		−1.846	1.111	0.016
ENSSSCG00000018563	SNORA48	small nucleolar RNA, H/ACA box 48 [Source:HGNC Symbol;Acc:HGNC:32641]	12	H/ACA	28S rRNA	−0.983	3.404	0.016
ENSSSCG00000018699		NULL	7	na		−0.965	2.744	0.016
ENSSSCG00000031232	SNORD60	Small nucleolar RNA, C/D box 60 [Source:HGNC Symbol;Acc:HGNC:10217]	3	C/D	28S rRNA	−1.096	1.538	0.017
ENSSSCG00000018462	SNORD65	Small nucleolar RNA, C/D box 65 [Source:HGNC Symbol;Acc:HGNC:32726]	12	C/D	18S rRNA	−1.125	1.220	0.018
ENSSSCG00000018650	SNORD56	Small nucleolar RNA, C/D box 56 [Source:HGNC Symbol;Acc:HGNC:10206]	17	C/D	18S rRNA	−1.010	2.227	0.018
ENSSSCG00000033121	SNORD38B	Small nucleolar RNA, C/D box 38B [Source:HGNC Symbol;Acc:HGNC:30356]	6	C/D	28S rRNA	−1.904	−0.165	0.021
ENSSSCG00000018934	SNORD61	Small nucleolar RNA, C/D box 61 [Source:HGNC Symbol;Acc:HGNC:10218]	X	C/D	18S rRNA	−1.432	1.263	0.023
ENSSSCG00000023906		NULL	6	scaRNA		−1.593	1.127	0.025
ENSSSCG00000031725	SNORD14	Small nucleolar RNA SNORD14 [Source:RFAM;Acc:RF00016]	9	C/D	18S rRNA	−0.801	2.453	0.025
ENSSSCG00000018591	SNORD105	Small nucleolar RNA, C/D box 105 [Source:HGNC Symbol;Acc:HGNC:32769]	2	C/D	18S rRNA	−1.380	0.193	0.025
ENSSSCG00000018494	SNORD83	Small nucleolar RNA SNORD83 [Source:RFAM;Acc:RF00137]	7	C/D	unknown	−1.085	0.913	0.032
ENSSSCG00000020093		NULL	1	scaRNA		−1.492	1.091	0.036
ENSSSCG00000018553		NULL	3	na		−1.787	0.374	0.036
ENSSSCG00000021904	SNORA5A	Small nucleolar RNA, H/ACA box 5A [Source:HGNC Symbol;Acc:HGNC:32588]	18	H/ACA	18S rRNA	−1.134	3.360	0.037
ENSSSCG00000038146	SNORD9B	Small nucleolar RNA, C/D box 49B [Source:HGNC Symbol;Acc:HGNC:32721]	12	C/D	U6 snRNA	−1.218	0.648	0.037
ENSSSCG00000018868	SNORD87	Small nucleolar RNA, C/D box 87 [Source:HGNC Symbol;Acc:HGNC:32746]	4	C/D	28S rRNA	−1.111	0.973	0.038
ENSSSCG00000018501	SNORA72	Small nucleolar RNA SNORA72 [Source:RFAM;Acc:RF00139]	4	H/ACA	5.8S rRNA	−1.179	2.325	0.041
ENSSSCG00000034185	SNORA73	Small nucleolar RNA SNORA73 family [Source:RFAM;Acc:RF00045]	6	H/ACA	unknown	0.838	1.528	0.041
ENSSSCG00000019634	SNORA49	Small nucleolar RNA, H/ACA box 49 [Source:HGNC Symbol;Acc:HGNC:32642]	14	H/ACA	unknown	−0.894	1.765	0.041
ENSSSCG00000019629	SNORA5C	Small nucleolar RNA, H/ACA box 5C [Source:HGNC Symbol;Acc:HGNC:32590]	18	H/ACA	18S rRNA	−1.178	1.187	0.043
ENSSSCG00000019738	SNORA79	Small nucleolar RNA, H/ACA box 79 [Source:HGNC Symbol;Acc:HGNC:32665]	7	H/ACA	U6 snRNA	−2.214	0.173	0.044

^a^, Ensembl (https://m.ensembl.org/index.html, accessed on 8 February 2023) gene ID. ^b^, Gene symbol. ^c^, Chromosome or scaffold number where gene is located. ^d^, Family type of snoRNAs. C/D box and H/ACA box snoRNAs. scaRNA, small Cajal body-associated RNAs. na, snoRNA predicted by RFAM and miRBase and family type is unknown. ^e^, Potential target RNAs reported in human orthologs in snOPY database (http://snoopy.med.miyazaki-u.ac.jp/snorna_db.cgi, accessed on 8 February 2023). ^f^, Log2 fold change relative to BCB+ oocytes. ^g^, Average expression levels, shown in TMM-normalised log2 count per million (CPM) sequence reads. ^h^, Significant difference in expression between BCB− and BCB+ oocytes by FDR test (*p* < 0.05).

**Table 3:** Longitudinal DEG analysis of the effect of MT supplementation during oocyte to blastocyst transition. Top 15 genes were short listed from Tables S6 and S7.

gene_id ^a^	Symbol ^b^	Description	Chr/Scaffold ^c^	logFC ^d^	logCPM ^e^	FDR ^f^
*Only in oocyte vs MT− balstocyst DEGs*						
ENSSSCG00000018070	tRNA-Trp	product=tRNA-Trp	MT	8.098	−2.836	9.01 × 10^−17^
ENSSSCG00000018073	tRNA-Cys	product=tRNA-Cys	MT	8.414	−2.516	1.32 × 10^−16^
ENSSSCG00000018071	tRNA-Ala	product=tRNA-Ala	MT	7.828	−3.004	2.39 × 10^−16^
ENSSSCG00000018062	tRNA-Val	product=tRNA-Val	MT	9.163	−0.652	1.30 × 10^−15^
ENSSSCG00000018072	tRNA-Asn	product=tRNA-Asn	MT	6.784	−3.061	2.33 × 10^−12^
ENSSSCG00000050090		NULL	15	6.195	−2.580	1.87 × 10^−6^
ENSSSCG00000003523	EPHA8	EPH receptor A8 [Source:VGNC Symbol;Acc:VGNC:87735]	6	5.328	−2.925	2.35 × 10^−6^
ENSSSCG00000045445		NULL	11	5.320	−2.805	6.20 × 10^−6^
ENSSSCG00000000439	KIF5A	kinesin family member 5A [Source:VGNC Symbol;Acc:VGNC:89472]	5	4.870	−2.845	1.34 × 10^−5^
ENSSSCG00000035495	KITLG	KIT ligand [Source:VGNC Symbol;Acc:VGNC:98061]	5	2.082	6.300	1.39 × 10^−5^
ENSSSCG00000001750	PAQR8	progestin and adipoQ receptor family member 8 [Source:VGNC Symbol;Acc:VGNC:91178]	7	3.598	2.226	1.41 × 10^−5^
ENSSSCG00000026861	NECTIN4	nectin cell adhesion molecule 4 [Source:VGNC Symbol;Acc:VGNC:90664]	4	5.308	−2.341	2.49 × 10^−5^
ENSSSCG00000004390	SESN1	sestrin 1 [Source:VGNC Symbol;Acc:VGNC:92753]	1	1.288	5.758	3.17 × 10^−5^
ENSSSCG00000027372	SAMD9	sterile alpha motif domain containing 9 [Source:HGNC Symbol;Acc:HGNC:1348]	9	4.991	−2.977	6.06 × 10^−5^
ENSSSCG00000008765	PCDH7	protocadherin 7 [Source:NCBI gene (formerly Entrezgene);Acc:100520035]	8	4.794	−3.279	7.96 × 10^−5^
*Only in oocyte vs MT+ balstocyst DEGs*						
ENSSSCG00000048420		Novel gene	AEMK02000602.1	6.819	0.974	2.76 × 10^−12^
ENSSSCG00000041004		Novel gene	4	5.747	−3.428	4.61 × 10^−10^
ENSSSCG00000018084	ND3	mitochondrially encoded NADH:ubiquinone oxidoreductase core subunit 3 [Source:VGNC Symbol;Acc:VGNC:99794]	MT	1.995	10.371	1.78 × 10^−9^
ENSSSCG00000043677		NULL	4	6.122	−2.129	3.82 × 10^−9^
ENSSSCG00000039862	TRIB3	tribbles pseudokinase 3 [Source:VGNC Symbol;Acc:VGNC:95791]	17	4.254	1.509	5.51 × 10^−9^
ENSSSCG00000028913		Novel gene	X	5.950	−3.147	9.18 × 10^−9^
ENSSSCG00000039947	KCNJ2	potassium inwardly rectifying channel subfamily J member 2 [Source:VGNC Symbol;Acc:VGNC:89357]	12	6.374	−2.696	1.50 × 10^−8^
ENSSSCG00000042056		NULL	6	5.426	−3.168	2.14 × 10^−8^
ENSSSCG00000049454		Novel gene	1	5.488	−2.866	2.71 × 10^−8^
ENSSSCG00000004673	SLC28A2	solute carrier family 28 member 2 [Source:VGNC Symbol;Acc:VGNC:93042]	1	6.691	−2.302	3.34 × 10^−8^
ENSSSCG00000031101	METTL21C	methyltransferase like 21C [Source:VGNC Symbol;Acc:VGNC:90159]	11	5.905	−3.068	6.28 × 10^−8^
ENSSSCG00000027387	MS4A12	membrane spanning 4-domains A12 [Source:VGNC Symbol;Acc:VGNC:90408]	2	5.392	−2.752	7.14 × 10^−8^
ENSSSCG00000047877		Novel gene	13	5.268	−3.333	7.82 × 10^−8^
ENSSSCG00000002867	CEBPG	CCAAT enhancer binding protein gamma [Source:VGNC Symbol;Acc:VGNC:86534]	6	2.065	5.153	1.49 × 10^−7^
ENSSSCG00000050874		NULL	AEMK02000598.1	5.882	−2.807	1.50 × 10^−7^

^a^, Ensembl (https://m.ensembl.org/index.html, accessed on 8 February 2023) gene ID. ^b^, Gene symbol. ^c^, Chromosome or scaffold number where gene is located. ^d^, Log2 fold change relative to BCB+ oocytes. ^e^, Average expression levels, shown in TMM-normalised log2 count per million (CPM) sequence reads. ^f^, Significant difference in expression between BCB+ oocytes and blastocysts by FDR test (*p* < 0.05).

## Data Availability

The datasets supporting the conclusions of this article are available in the NCBI Sequence Read Archive (https://www.ncbi.nlm.nih.gov/sra, accessed on 8 February 2023) under the two BioProject IDs PRJNA429045 and PRJNA777282.

## Supplementary Materials

The following supporting information can be downloaded at: https://www.mdpi.com/article/10.3390/ijms24043783/s1.
