# Supplementary material for: Mitochondrial DNA Deficiency and Supplementation in Sus scrofa Oocytes Influence Transcriptome Profiles in Oocytes and Blastocysts
Source: Int J Mol Sci. 2023 Feb 14;24(4):3783. doi: 10.3390/ijms24043783 (PMC9963854; doi:10.3390/ijms24043783)
Supplement: Supplementary file 1 [file ijms-24-03783-s001.zip › ijms-2209386 Sup Figure final.pdf]

Supplementary Figures

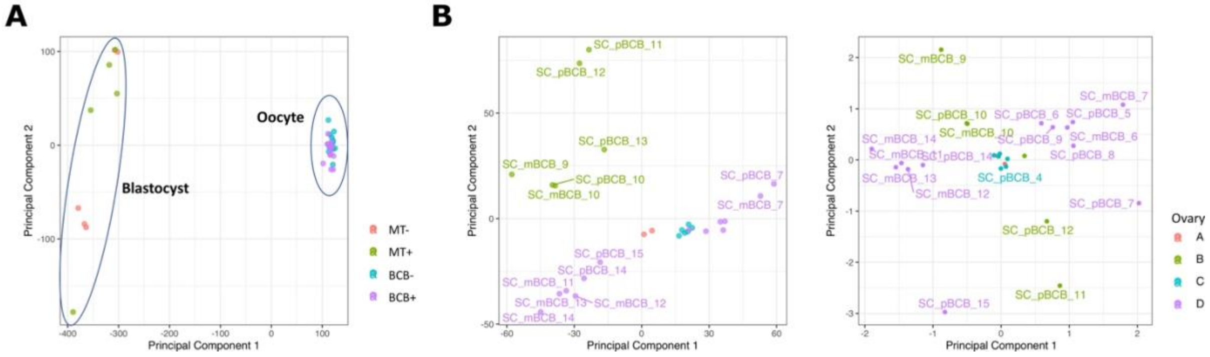

Figure S1. Principal component analysis (PCA) of single oocyte and blastocyst RNAseq data. (**A**) PCA plot for all RNAseq data used in this study, BCB+ and BCB− oocytes and blastocysts with (MT+) or without (MT−) mtDNA supplementation. (**B**) Single oocyte data are grouped and indicated by different colours for source of original ovary A to D (left panel), indicating effect of ovary source on RNAseq profile. PCA after batch effect correction using ovary source as a covariate (right panel).

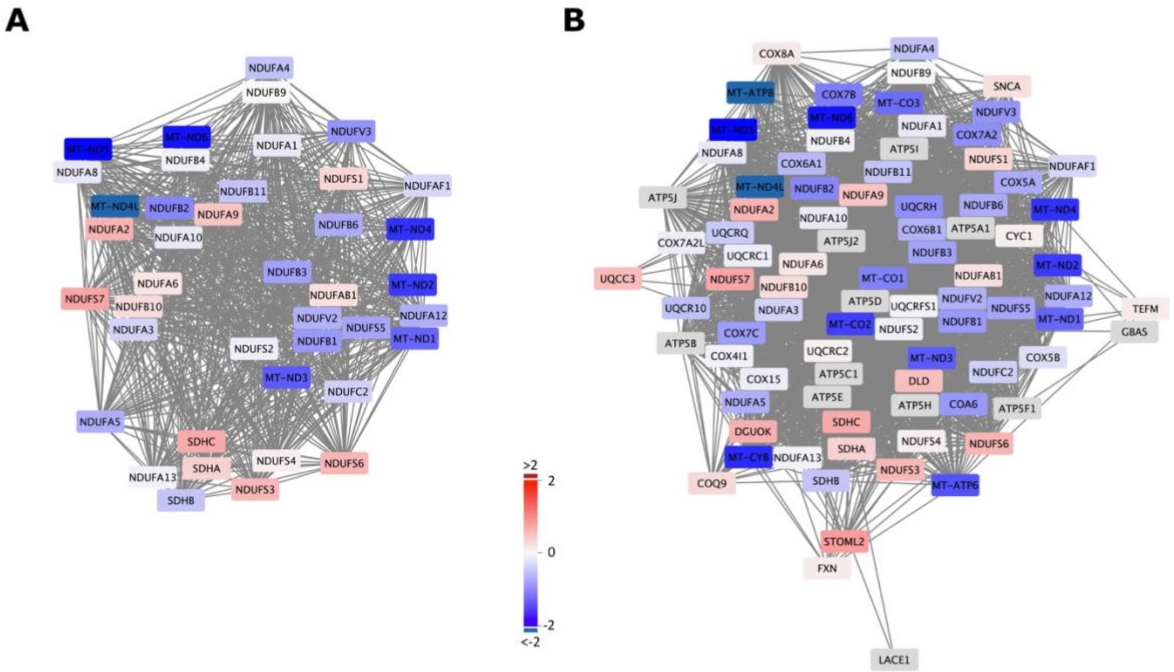

Figure S2. Potential protein interactions of the identified DEGs involved in OXPHOS (**A**) electron transport chain complexes I and II and (**B**) all the related proteins. Levels of differential gene expression between BCB+ and BCB− oocytes are shown as fold change by colour scale. Red and blue represent up- and down-regulation in BCB− oocytes. Genes in grey box have no DEG data due to low or no expression.

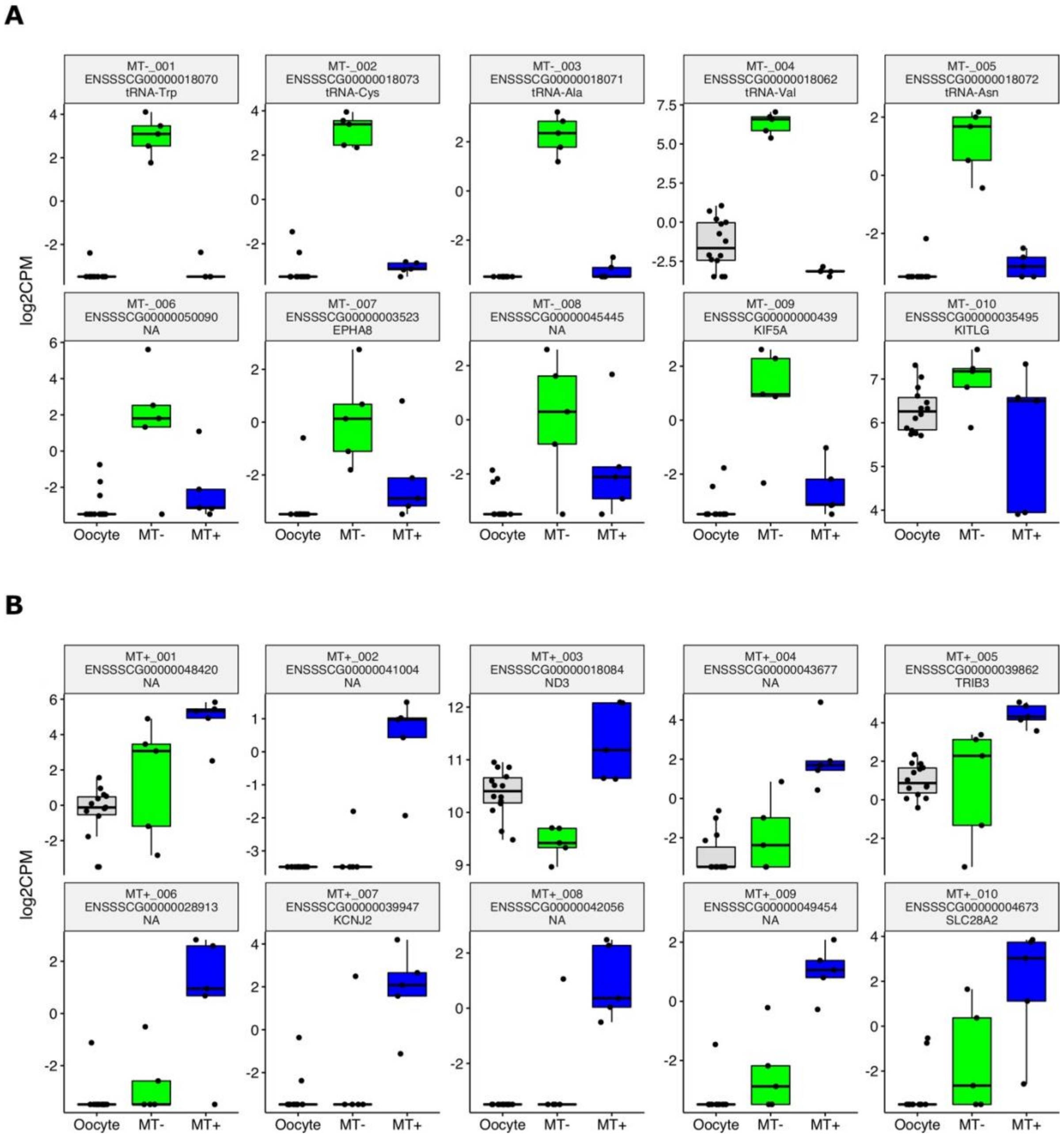

Figure S3. Subsets of DEGs identified in one of the oocyte to blastocyst transition processes by longitudinal analysis. (**A**) Top 10 DEGs uniquely identified in oocyte to MT− blastocyst transition (Table S6) are presented individually by jittered-boxplot. (**B**) Top 10 DEGs uniquely identified in oocyte to MT+ blastocyst transition (Table S7). Levels of expression were plotted on the *y*-axis as log2CPM value.
